# Influence of autochthonous *Lactiplantibacillus plantarum* strains on microbial safety and bioactive compounds in a fermented quinoa-based beverage as a non-dairy alternative

**DOI:** 10.1016/j.fochx.2025.102294

**Published:** 2025-02-21

**Authors:** Pamela Canaviri-Paz, Thamani Freedom Gondo, Anna Kjellström, Tawanda Mandoga, Jaison Sithole, Elin Oscarsson, Margareta Sandahl, Åsa Håkansson

**Affiliations:** a Department of Process and Life Science Engineering, Faculty of Engineering, LTH, Lund University. Box 124, SE-221 00 Lund, Sweden; b Centre for Analysis and Synthesis, Department of Chemistry, Faculty of Science, Lund University. Box 124, 221 00 Lund, Sweden

**Keywords:** Starter cultures, Native microbiota, *Enterobacteriaceae*, Phenolic compounds, Saponins, High-resolution mass spectrometry, Next-generation sequencing

## Abstract

Plant-based alternatives are considered microbiologically safe; however, recent studies have raised concerns about hygienic quality. Additionally, the relationship between microbiological safety and polyphenolic content in fermented products remains unexplored. This study assessed the potential of four autochthonous *Lactiplantibacillus plantarum* strains (3, 5, 9, and 10) to impact microbial composition and modulate polyphenol and saponin levels in a quinoa-based beverage. The results identified *Lactiplantibacillus plantarum* strains 3, 9, and 10 as effective in inhibiting *Enterobacteriaceae* (*p* = 0.001), and increasing concentrations of glycosylated flavonoids, 3-phenyllactic acid, and saponins. However, *Lactiplantibacillus plantarum* 10 demonstrated a decrease in saponin levels, whereas *Lactiplantibacillus plantarum* 5 increased the abundance of aglycones, highlighting strain-specific differences. Notably, principal component analysis revealed less differences between inoculated samples and control, indicating potential contribution of the native microbiota to the fermentation. This study enhances the understanding of interactions between starter cultures, native microbiota, and bioactive compounds in plant-based fermented beverages.

## Highlights

• The initial microbiome of quinoa is dominated by Proteobacteria, but fermentation encourages the growth of Firmicutes.
• NGS and viable counts showed variations in *Enterobacteriaceae* with different strains.
• Most changes in phenolic and saponin content were attributed to the action of native microorganisms.
• *L. plantarum* 3 and 9 displayed elevated glycosylated flavonoid content after fermentation.
• *L. plantarum* 5 increased aglycone abundance, while L. *plantarum* 10 decreased saponin content.

## 1 Introduction

The market offers a diverse range of products derived from various plant matrices to meet consumers' growing demand for healthy and sustainable alternatives. To serve as effective substitutes for animal- and dairy-based foods, these products must contain comparable levels of nutrients and bioactive compounds (Klapp, Feil, & Risius, 2022). To improve the bioavailability of nutrients in plant-based products microbial fermentation is commonly used (Rollán, Gerez, & Leblanc, 2019). Although fermentation is generally seen as enhancing safety, doubts remain about the effectiveness of current methods and standards for ensuring microbiological safety in fermented beverages (Kain, Albahri, Plötz, & Jessberger, 2024).

Previous research has explored quinoa-based foods fermented by lactic acid bacteria (LAB) (Cerdá-Bernad, Valero-Cases, Pastor, Frutos, & Pérez-Llamas, 2021). These studies have revealed several beneficial characteristics, including high nutritional value (Zhang et al., 2021), elevated polyphenolic content (Rizzello et al., 2017), and reduced saponin levels (Bolívar-Monsalve, Ceballos-González, Ramírez-Toro, & Bolívar, 2018). Most of the studies suggest that the alterations in polyphenolic and saponin compounds are primarily due to the enzymatic activities of the bacterial strains participating in the fermentation process.

While quinoa seeds are generally considered to have minimal native microbiota and are thought to be less susceptible to pathogenic contamination due to their low moisture content, recent studies using culture-dependent techniques have begun to characterize the microbiota present in quinoa seeds and flour. The studies have identified microorganisms from the Firmicutes phylum, including *Levilactobacillus brevis* and *Lactiplantibacillus plantarum,* as well as from the Proteobacteria phylum such as *Enterobacter cloacae, Enterococcus gallinarum,* and *Klebsiella michiganensis* (Canaviri-Paz, Oscarsson, & Håkansson, 2021), of which the latest are known to be potential pathogens and carriers of antibiotic resistance. Furthermore, Ortuño and co-workers analysed different parts of the quinoa plant, including the seeds, and found *Bacillus subtilis, Bacillus pumilus,* and *Bacillus amilequefasciens* (Ortuño, Claros, Gutiérrez, Angulo, & Castillo, 2014). In our previous study, several viable cells of *Enterococcus* spp.*, and Pediococcus* spp.*,* were also detected after pasteurization and fermentation of the quinoa seeds (Canaviri Paz, Janny, & Håkansson, 2020). The presence of pathogenic microorganisms during the manufacturing process can disrupt fermentation and pose potential health risks.

The microbiological quality of fermented food products relies on potent starter cultures (Valerio et al., 2020) and using lactic acid bacteria (LAB) from plant origins has proven effective in ensuring safety and enhancing functionality (Torres, Verón, Contreras, & Isla, 2020). However, the early interactions between native microbiota and starter cultures, as in quinoa fermentation, where native microbes temporarily influence bioactive compounds like polyphenols, are not yet fully understood. Various phenolic compounds and saponins are present in quinoa seeds (Gómez-Caravaca, Segura-Carretero, Fernández-Gutiérrez, & Caboni, 2011), with a fraction of these bioactive molecules being integrated into the plant's cell wall structure. To increase the number of free polyphenols in quinoa, acids, bases, or enzymes such as pectinase, xylanase, and feruloyl esterase have been used (Z. Wang, Li, Ge, & Lin, 2020). Besides it has been demonstrated that pathogenic bacteria possess resistance to oxidative stress and might therefore release or degrade polyphenolic compounds during fermentation (Z. Wang et al., 2020). The phenolic composition in fermented quinoa has primarily focused on total phenolics and antioxidant activity (Melini & Melini, 2021; Rizzello et al., 2017), typically measured through colorimetric methods. Although compounds such as saponins are also of interest due to their effect on overall quality and consumer acceptance. The fermentation process commonly leads to increased antioxidant activity, mainly due to the release of phenolic compounds previously bound to protein and carbohydrates in the quinoa matrix (Melini & Melini, 2021; Starzyńska-Janiszewska et al., 2023). While comprehensive metabolite profiles of unfermented quinoa have been established (Gómez-Caravaca et al., 2011; Tang et al., 2015), there remains a significant gap in understanding how the native microbiota and changes in phenolic content influence the safety and functionality of fermented quinoa, and studies identifying individual bioactive compounds in fermented quinoa are still limited. Similar analysis and identification of polyphenols and saponins should therefore be developed for a fermented quinoa-based beverage, to understand the changes in those secondary metabolites.

A previous study isolated four autochthonous *Lactiplantibacillus plantarum* (*Lpb*. *plantarum*) strains from white quinoa grains during spontaneous fermentation (Canaviri-Paz, Oscarsson, & Håkansson, 2021).Two strains (*Lpb. plantarum* 3 and 10) can degrade xylose, while the others (*Lpb. plantarum* 5 and 9) lack this ability. All strains demonstrated tannin-degrading properties, suggesting they could enhance the functionality of the fermented quinoa-based beverage by modulating polyphenolic and saponin compounds. This study aimed to assess the ability of the native *Lpb*. *plantarum* strains to modify the microbiota of a quinoa-based beverage, prevent growth of potential pathogens, and evaluate their impact on polyphenol and saponin content. This dual focus bridges a significant knowledge gap in fermented plant-based products. By evaluating the specific strains, the research highlights the strain-dependent effects on microbial inhibition and bioactive compound profiles, offering targeted insights for strain selection in functional food development.

## 2 Materials and methods

### 2.1 Chemicals and reagents

Chemicals and reagents used during the study were: Formic acid (98 %, purity p.a., ACS, Merck, Darmstadt, Germany), acetonitrile (99.9 %, LC/MS Gradient Grade, VWR, Pennsylvania, USA), and methanol (99.9 %, LC/MS-Gradient Grade, VWR, Pennsylvania, USA). Polyphenols used as standards were purchased from Sigma Aldrich (St. Louis, MO, USA) as follows: Gallic acid (GA), 3,4-dihydroxybenzoic acid (3,4-DHBA), 4-hydroxybenzoic acid (4-HBA), chlorogenic acid, vanillic acid, syringic acid, epicatechin, cinnamic acid, catechin, caffeic acid, p-coumaric acid, ferulic acid, rutin, quercetin 3-O-glucoside, mycetrin, quercetin, kaempferol. Stock solutions of each external standard were prepared at a concentration of 1000 mg/L in methanol LC/MS grade.

Previously isolated and characterized autochthonous *Lpb*. *plantarum* strains (Canaviri-Paz, Oscarsson, & Håkansson, 2021), were used as starter cultures at the concentrations of 7.12 log10 cfu/mL of *Lpb. plantarum* 3, 7.04 log10 cfu/mL of *Lpb. plantarum* 5, 7.03 log10 cfu/mL of *Lpb. plantarum* 9, and 6.90 log10 cfu/mL of *Lpb. plantarum* 10. Reactivation of cells is described in supplementary material.

Metrohm 744 pH meter (Metrohm Ltd., Herisau, Switzerland) was calibrated according to the manufacturer's recommendation. The concentrations of D-(−)-, and L-(+)- enantiomers were measured using an Enzytech D/L lactic acid kit (R-Biopharm Darmstadt, Germany). A microplate reader (SPECTROstart^Nano^, BMG LABTECH, Germany) was used to measure the concentration of the lactates. As culturing media, Violet red bile dextrose agar (VRBD, Merck, Germany), Tryptic soy agar (TSA, Fluka Missouri, USA) and Rogosa agar (Oxoid) were used. Anaerobic gas packs (BBl, Becton Dickinson and Company, USA) were used to create the anaerobic conditions. Peptone water solution (NaCl, Merck, Germany, 8.5 g/L; Bacteriological peptone, Oxoid, 1 g/L) was prepared, autoclaved at 121 °C for 15 min and cooled at 4 °C overnight before use.

For next-generation sequencing (NGS) analysis, the following equipment and consumables were needed: The Illumina MiSeq system (Illumina, San Diego, CA, USA), lysis buffer (SL-1 buffer, Macherey-Nagel GmbH &Co. KG, Düren, Germany), NucleoSpin Soil Kit ((Macherey-Nagel GmbH &Co. KG, Düren, Germany), and purification kit AMPure XP beads (Agencourt, Beckman coulter genomics, Germany), all used following the manufacturer's instructions.

Tap water was initially autoclaved at 121 °C for 15 min and cooled down to 4 °C overnight. Glass bottles of 1 L with cap (IKEA, Sweden, 302.135.52) were previously washed and sterilized at 121 °C for 15 min. An electrical thermometer (multi-thermometer), a blender (Electrolux, Great Blending TruFlow™blades, ESB5400BK) and a juice strainer (Jonas of Sweden, Lindén International AB) were used for the preparation of the quinoa-based beverage.

### 2.2 Preparation of the fermented quinoa-based beverage

Desaponified white quinoa seeds imported from Bolivia (Saltå Kvarn AB, Stockholm, Sweden) acquired from a local supermarket in Lund, Sweden, were used for beverage preparation. The preparation, as previously described by Canaviri-Paz et al. (Canaviri-Paz et al., 2021), is shortly described below. Initially, the seeds were efficiently washed for 30 min in water (1:3; *w*/*v*), replacing the water every 15 min and irrigating the grains in between to ensure the elimination of possible impurities. The washing procedure was performed until the water became clear and foamless. The quinoa grains were dried at temperatures between 180 °C to 186 °C on a stove (Elektro Ԑ Helios) for approximately 15 min with constant agitation and then roasted at 144 ± 3 °C for 25 ± 2 min. The roasted quinoa grains were cooled down at room temperature for at least 30 min before being mixed. A proportion of roasted quinoa: tap water of 1:8 (*w*/*v*) was homogenized using a blender. The mixture was filtered, collected in glass bottles, and inoculated with the individual strains. Three inoculated bottles per bacterium and one without inoculum used as control, marked as X, were incubated at 30 °C for 48 h. The fermented quinoa-based beverages were then stored in cold conditions at 4 °C for 28 days. The sampling points were at time 0 (before fermentation), after 2 days of fermentation (48 h) and after 14 and 28 days of cold storage. Aliquots of 15 mL were withdrawn in triplicate per bottle and grouped according to the experimental procedure. The microbiological analysis was performed immediately after sampling. The remaining duplicates were stored in darkness at −20 °C until further analyses.

### 2.3 pH and acidity measurement

The pH was measured at each sampling point. The acidity was measured following the manufacturer's instructions with minor modifications. Briefly, aliquots of 5 mL of quinoa-based beverage were centrifuged at 4266 ×*g* for 15 min and the supernatant was collected. An aliquot of 1 mL from the supernatant was diluted (1:100) and used according to the manufacturer recommendation for the Enzytech D/L lactic acid kit. The absorbance of 260 μL of the diluted samples was measured in triplicates at 320 and 340 nm by the microplate reader.

### 2.4 Quantification of viable cells

Aliquots of 10 mL of quinoa beverage were withdrawn and immediately diluted in 90 mL of sterile bacteriological peptone water. Consequently, a conventional ten-fold dilution series was performed and 100 μL of the final dilution was spread plated in duplicates on Rogosa agar, incubated anaerobically at 37 °C for 72 h (lactobacilli), VRBD incubated aerobically at 37 °C for 24 h (*Enterobacteriaceae*), and TSA incubated aerobically at 30 °C for 72 h (total aerobic count). Viable cell counts were determined using plates with 20 to 200 colonies.

### 2.5 Next generation sequencing

Aliquots of 15 mL of the fermented quinoa-based beverage were thawed and centrifuged at 6300 ×*g* for 20 min. The pellet was resuspended in SL1-buffer and the mixture was used to extract the DNA using a NucleoSpin Soil kit. Identification of bacterial taxa was achieved following the 16S Metagenomics Sequencing Library Preparation protocol based on a two-step process. In the first step, thermal cycling was performed in an Eppendorf MasterCycler (Eppendorf, Hamburg, Germany) targeting the regions V3-V4. The paired primers 341F (5′- TCG TCG GCA GCG TCA GAT GTG TAT AAG AGA CAG CCT ACG GGN GGC WGC AG −3′), and 805R (5′- GTC TCG TGG GCT CGG AGA TGT GTA TAA GAG ACA GGA CTA CHV GGG TAT CTA ATC C – 3′) were used, resulting in fragments of 550 bp. The products were purified using the AMPure XP beads. A second thermal cycling reaction was then performed to attach indexes (Nextera XT index kit, Illumina) to the amplified fragments. The products were purified as described above. The concentration of the resulting DNA fragments was determined using a Qubit 4.0 Fluorometer (Thermofisher Scientific, Sweden), after which the samples were combined in equimolar ratios to a final concentration of 6 pMol. The fragments were sequenced using the Illumina MiSeq platform (Illumina, USA) with the MiSeq reagent kit V3 (Illumina Inc., San Diego, USA) and a read length of 2 × 300 bp paired-end sequencing following the manufacturer's recommendations.

### 2.6 Extraction and analysis of polyphenols

#### 2.6.1 Extraction of polyphenols and saponins

Samples of 15 mL were thawed at 4 °C overnight in darkness. The extraction was performed following a previously published protocol (Gondo, Huang, Marungruang, Heyman-Lindén, & Turner, 2024), with some modifications. Aliquots of 5 mL (*n* = 3 per bacterial strain; *n* = 1 per control) were centrifuged at 4266 ×*g* for 15 min and the supernatant was collected and stored at 4 °C (in darkness in closed tubes). To the residue, 5 mL of 1 % formic acid in methanol was added. The samples were then vortexed for 30 s and left for 24 h at 4 °C in darkness for extraction. The samples were vortexed for one min and centrifuged at 4266 ×*g* for 20 min. The supernatant was collected and mixed with the previous aqueous portion. The final volume was homogenized to 10 mL with 1 % formic acid in Milli-q water, in a 10 mL volumetric flask. About 1.5 mL of the solution was filtered through a PTFE membrane of 0.2 μm pore diameter (VWR, Pennsylvania, USA) and collected in a 2 mL amber vial (9 mm, Amber 51w/Patch+PTFE/Silicone/PTFE, Phenomenex, United States), prior to HPLC analysis.

#### 2.6.2 High-performance liquid chromatography

The analysis of polyphenols was conducted following the method modified from literature (Gondo, Jönsson, Karlsson, Sandahl, & Turner, 2023). An Agilent Technologies high-performance liquid chromatography (HPLC) 1100 series from Agilent (Santa Clara, California, USA) was used, equipped with a G1322A degasser, a G1376A quaternary gradient solvent pump, a G1377A microwell plate sampler, a G1316A thermostatted column compartment, a G1315B diode array detector (DAD) and a ChemStation software. A fused core Xselect C18 column (150 mm length × 3 mm diameter, 3.5 μm particle size) from Waters (Milford, USA), and an eluent consisting of a binary mixture of solvent A (1 % formic acid in Milli-Q water) and solvent B (1 % formic acid in acetonitrile) were used. Gradient elution according to the following program was used: 0 to 5 min isocratic elution with 5 % B, 5 to 10 min (5–10 % B), 10 to 15 min (10–15 % B), 15 to 20 min (15–20 % B), 15 to 18 min (20–30 % B), 18 to 23 min (30–40 % B), and ultimately 23 to 30 min (40–80 % B). Additionally, 2 min of post-run time was employed using the initial gradient composition. A column temperature of 30 °C, a flow rate of 0.5 mL/min and an injection volume of 10 μL per sample were used. UV/VIS signals at 280 and 360 nm were acquired for detection of phenolic compounds and flavonoids respectively, while 325 nm was used for measuring chlorogenic acid, ferulic acid and p-coumaric acid. External standards were used for making calibration curves which were used for quantification of the phenolic compounds. The standards were analysed in triplicates and the average values of the absorbance were plotted against the concentration in the range of 2–200 μg/mL. Limits of detection (LOD) and quantification (LOQ) were calculated from the calibration curve using equations; 3.3 × Sy/S and 10 × Sy/S respectively, where Sy corresponds to the standard deviation of the response, and S is the slope of the calibration curve in the range 0.2–30 μg/mL.

#### 2.6.3 Ultrahigh-performance liquid chromatography/mass spectrometry

The above HPLC method was transferred to an ultra-high performance liquid chromatography /quadrupole time-of-flight - mass spectrometry (UHPLC/QTOF-MS) system, of which the settings were based on an online Waters calculator (Waters, 2019). The UHPLC methods were as follows: The mobile phase consisted of (A) water with 0.1 % formic acid and (B) acetonitrile with 0.1 % formic acid. Based on the online Waters calculator, the applied gradient was as follows: 0–1.73 min (5 % B); 1.73–3.35 min (5–20 % B); 3.35–4.97 min (20 % B); 4.97–5.94 min (20–25 % B); 5.94–6.59 min (25 % B); 6.59–7,56 min (25–70) 7.56–8.21 min (70 % B) and 8.21–14.68 min (70–100 % B). A flow rate of 0.4 mL/min and a column temperature of 55 °C were used. The column was a Waters ACQUITY UPLC CSH C18 100 × 2.1 mm, 1.7 μm particle size and an injection volume of 3 μL was set. A mass spectrometer (XEVO-G2 QTOF) with electrospray ionization (Waters, MS Technologies, Manchester, UK) was employed with the following settings: negative acquisition mode, mass range (*m*/*z*) was 100–1200 Da; capillary voltage, 2.5 kV; cone voltage, 40 V; source temperature, 120 °C; desolvation temperature, 500 °C; cone gas flow rate, 50 L/h; and desolvation gas flow rate, 700 L/h. MS/MS data was acquired through data dependence acquisition employing collision energy range between 15 and 65 kV. MS-DIAL (Ver 4.9.221218) was used for MS data deconvolution and treatment. The annotation of peaks was carried out through the help of various databases in MS-DIAL (i.e. Fihn/Vaniya natural product library, RIKEN PlasMA bio-MS/MS from plant tissues, Metabobase and ReSpect), as well as through the support of literature (Gómez-Caravaca et al., 2011; Kuljanabhagavad, Thongphasuk, Chamulitrat, & Wink, 2008). During data processing, MS1 mass tolerance was set to 0.01 Da, while MS2 tolerance was set to 0.025 Da. The minimum peak height was set to 1000 amplitude for peak detection.

### 2.7 Statistical analysis

The number of viable cells, pH, acidity, and HPLC generated data were statistically analysed using Sigma Plot version 14.0 (SYSTAT Software, Point Richmond, USA), applying Kruskal-Wallis One-Way Analysis of Variance (ANOVA) on Ranks or a Mann-Whitney Ranks Sum test when appropriate. Results were presented as Mean ± SD after passing the normality test Shapiro-Wilk and *p* values <0.050 were considered significant. Data generated from NGS was pre-processed using DADA2 in Qiime2 with Greengenes v13.5 as reference genomes. The data was statistically analysed in R version 3.6.3. excluding all the sequences not identified as bacteria. Calibration curves were calculated and graphed using Microsoft Excel 2010. Principal component analysis based on a graphical user interface developed for MATLAB (Ballabio, 2015), was used to evaluate the trend in the data generated from the MS. For the heatmap plot, the MS intensities of detected compounds were normalized to the maximum intensity for the respective compound, to show the trend of each compound against various species.

## 3 Results and discussion

### 3.1 Quantification of viable cells and composition of bacterial taxa

**Fig. 1:**
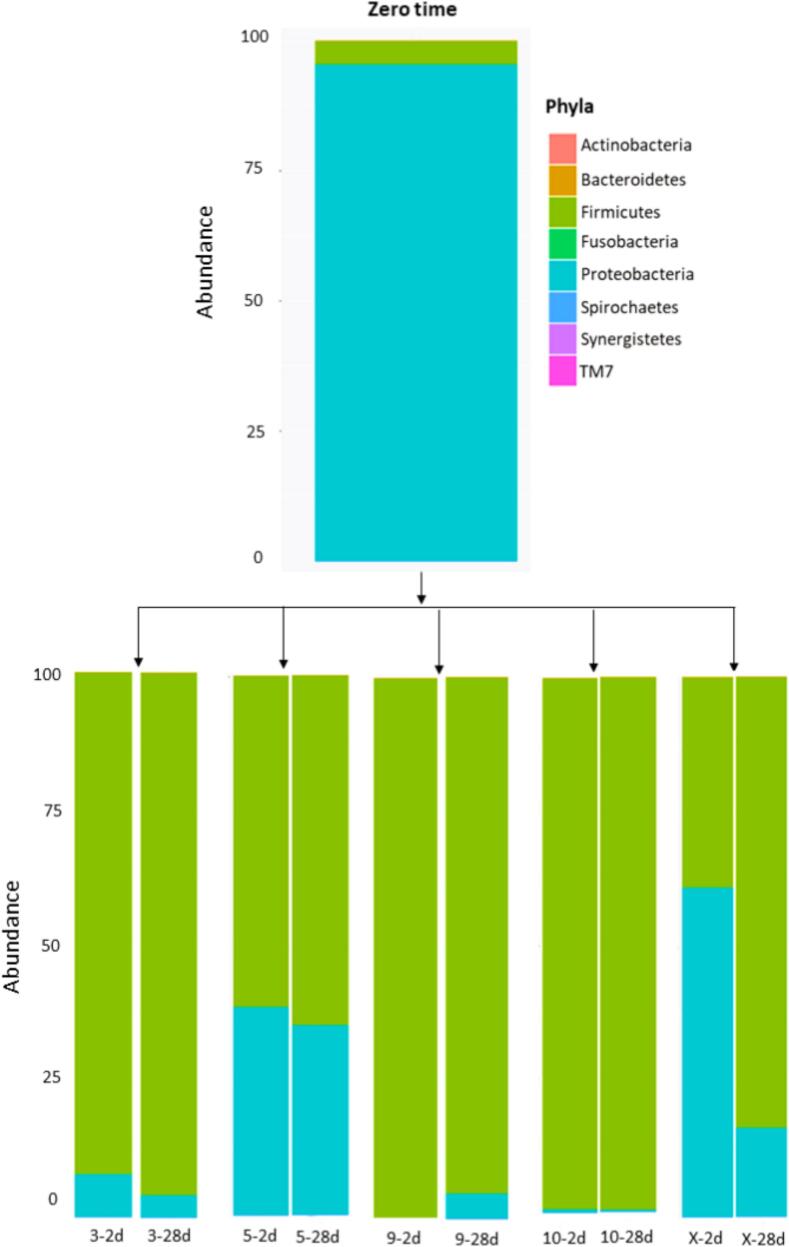
Relative phyla abundance before fermentation (zero time), after 2 days of fermentation, and at 28 days of storage time for *Lpb. plantarum* 3; 5; 9;10, and control (X).

In this study, quinoa-based beverages were individually fermented using four autochthonous *Lactiplantibacillus plantarum* strains isolated from quinoa seeds. The ability of these strains to inhibit potential pathogens was assessed using NGS and plate count methods. The bacterial composition of the non-fermented quinoa-based beverage was dominated by Proteobacteria, accounting for over 90 % of the microbial community, as indicated by the sequencing results (Fig. 1). Similar findings were reported by Cai et al. (Cai, Wang, Bhadra, & Gao, 2020) who found a dominance of the phylum Proteobacteria followed by Actinobacteria in quinoa plants. Those phyla include potential pathogens from the *Enterobacteriaceae* and *Pseudomonadaceae* families (Roch et al., 2024).

The results obtained from viable count (Table 1) on VRBD, Rogosa, and TSA agars showed that the effectiveness of the bacterial strains to dominate the microbiological niche during the fermentation depended on the strain itself. In the spontaneously fermented quinoa beverage used as control, the number of *Enterobacteriaceae* increased from a median of 2.69 ± 0.6 log10 cfu/mL to median of 7.43 ± 0.3 log10 cfu/mL (Table 1). The number of *Enterobacteriaceae* decreased significantly after 2 days of fermentation to undetectable values for *Lpb. plantarum* 3 (*p* = 0.001), *Lpb. plantarum* 9 (*p* = 0.002), and *Lpb. plantarum* 10 (p = 0.001). However, for *Lpb. plantarum* 5 viable counts of *Enterobacteriaceae* cells were still found (1.58 ± 1.7 log10 cfu/mL), but in comparison to the control, a statistically significant difference in *Enterobacteriaceae* count was found after 2 days of fermentation (p = 0.001), and after 14 days (p = 0.001) and 28 days (p = 0.001) of storage respectively (Table 1). The results indicate that while *Lpb. plantarum* 5 can survive and multiply during fermentation, it does not effectively inhibit the proliferation of *Enterobacteriaceae*. The presence of viable *Enterobacteriaceae* poses a microbiological risk and may negatively impact human health (Oscarsson, Hård Af Segerstad, Larsson, Östbring, & Agardh, 2020). In other plant-based beverages made from nuts, soybeans, and almonds *Enterobacteriaceae* was detected in 43 out of 138 products (Mukuna, Mafiz, Pokharel, Tobenna, & Kilonzo-Nthenge, 2021), highlighting the significance of this bacterial family in fermented products. Although plate counts showed no cell growth for *Lpb. plantarum* 3, *Lpb. plantarum* 9, and *Lpb. plantarum* 10, NGS detected a low percentage of Proteobacteria after 28 days of storage (Fig. 1). This result probably reflects NGS analysis of DNA from all cells, both living and dead (Jagadeesan et al., 2019).

**Table 1:** Viable cell count expressed as median ± SD log10 cfu/mL of fermented quinoa beverage.

		Native starter culture			
Time	Control (X)	*Lpb. plantarum* 3	*Lpb. plantarum* 5	*Lpb. plantarum* 9	*Lpb. plantarum* 10
(Days)	Median ± SD	Median ± SD	Median ± SD	Median ± SD	Median ± SD
***Enterobacteriaceae***					
0	2.69 ± 0.6^ab^	3.09 ± 0.3^ab^	2.80 ± 1.0^ab^	1.54 ± 0.3^ab^	2.70 ± 0.6^ab^
2	7.43 ± 0.3^ac^	<1^ac^	1.58 ± 1.7^ac^	<1^ac^	<1^ac^
14	7.49 ± 0.3^c^	<1^c^	2.53 ± 1.3^c^	<1^c^	<1^c^
28	2.96 ± 0.2^abc^	<1^c^	1.78 ± 1.0^c^	<1^c^	<1^c^
**Total aerobic count**					
0	4.79 ± 0.8^ab^	4.80 ± 0.1^ab^	4.00 ± 1.9^ab^	2.00 ± 1.2^ab^	2.92 ± 0.3^ab^
2	8.73 ± 1.2^abc^	9.18 ± 0.1^ab^	10.0 ± 0.8^ab^	10.3 ± 0.2^ac^	11.4 ± 0.1^ac^
14	9.06 ± 0.1^bc^	9.28 ± 0.1^b^	7.81 ± 0.3^b^	10.3 ± 0.7^c^	8.74 ± 0.4^a^
28	6.27 ± 0.1^c^	10.0 ± 0.3^c^	7.54 ± 0.4^b^	10.1 ± 2.8^c^	8.30 ± 0.4^c^
**lactobacilli**					
0	<1^ab^	<1^ab^	1.00 ± 0.7^ab^	<1^ab^	<1^b^
2	8.81 ± 1.0^ac^	12.2 ± 0.1^ac^	10.1 ± 3.8^ac^	11.3 ± 0.2^ac^	12.6 ± 0.7^ac^
14	7.91 ± 0.8^c^	11.3 ± 0.1^c^	11.1 ± 0.2^c^	9.64 ± 0.2^c^	11.7 ± 0.2^c^
28	9.0 ± 1.2^bc^	11.0 ± 0.2^c^	10.1 ± 0.4^c^	10.3 ± 0.8^b^	11.6 ± 1.1^c^

a Analyses of variance between values belonging to the same column considered statistically significant (*p* < 0.05).

b No statistically difference between values per row comparing the control group (*n* = 4) and the inoculated quinoa-based beverages (*n* = 3, individually per strain).

c Analyses of variance with statistically significance (p < 0.05) between the control group and the native starter cultures, corresponding to the values per row.

An increased relative abundance of the Firmicutes phylum and a multiplication of viable lactobacilli were detected after 2 days of fermentation (Table 1). The use of *Lpb. plantarum* 3 (12.2 ± 01 log10 cfu/mL), *Lpb. plantarum* 9 (11.3 ± 0.2 log10 cfu/mL), and *Lpb. plantarum* 10 (12.6 ± 0.7 log10 cfu/mL) altered over 85 % of the microbial community composition in the fermented quinoa-based beverages. No differences between the strains were observed, despite *Lpb*. *plantarum* 3 and *Lpb*. *plantarum* 10 possessing the capacity to degrade xylose, a major component of xylan naturally found in quinoa stalks (Canaviri-Paz, Oscarsson, & Håkansson, 2021; Gil-Ramirez et al., 2018). An increase of Firmicutes was also observed in samples inoculated by *Lpb. plantarum* 5 but to a lower percentage compared to the other *Lpb. plantarum* strains, and no further changes were observed during storage. In the control samples, Firmicutes increased by 40 % and continued to rise over the 28-day storage period (Fig. 1), indicating that the microbiota remains in a dynamic state throughout storage.

### 3.2 pH and acidity measurement

The pH was measured at every sampling occasion, i.e. 0, 2, 14 and 28 days. Both the control and inoculated beverages initially exhibited mildly acidic pH values, averaging 6.4. Following fermentation, a statistically significant decrease in pH (*p* = 0.002) was observed in all groups, with average pH values dropping to approximately 4 (Table 2). Similar results were obtained on a groupwise comparison between the inoculated quinoa-based beverages and control (*p* = 0.001) after 2 days of fermentation. At 14 and 28 days of storage, statistical differences between the inoculated beverages and the control were still found (p = 0.001 and *p* = 0.010) (Table 2). A reduction in pH is often regarded as indicative of microbial control during fermentation, however, this interpretation may overlook concerns regarding the microbial quality of fermented foods. Bacterial species within the Proteobacteria phylum, known to produce lactic acid, exhibit resistance to heat and acidic environments and are commonly found in plant-based matrices (Sakandar, Usman, & Imran, 2018). However, other organic acids produced during fermentation may also alter the pH and the overall taste of the beverage. Both *Lpb*. *plantarum* 5 and the control showed a decrease in pH, yet viable *Enterobacteriaceae* were identified in these samples. For pH to be used as a safety indicator of *Enterobacteriaceae* growth, it should decrease to below 4 (Canaviri Paz et al., 2020).

**Table 2:** pH and D-, L-lactic acid enantiomers concentration during fermentation of the quinoa-based beverage at different time points.

**Starter** **culture**	**Time** **(Days)**	**pH**		**Lactic acid (g/L)**			
				**D-(-)-**		**L-(+)-**	
		**Mean ± SD**	**p**	**Mean ±SD**	**p**	**Mean ± SD**	**p**
**Control (X)**	0	6.38 ± 0.11		-	-	-	-
	2	4.51 ± 0.18^e^	0.029^d^	-	-	-	-
	14	4.42 ± 0.23^f^	0.343	-	-	-	-
	28	4.86 ± 0.25^f^	0.486	-	-	-	-
***Lpb. plantarum* 3**	0	6.41 ± 0.01		0.00 ± 0.00		0.00 ± 0.00	
	2	3.46 ± 0.20^e^	0.002^d^	5.09 ± 0.64^g^	0.002^d^	7.24 ± 0.31^g^	0.002^d^
	14	3.45 ± 0.04^f^	0.093	5.70 ± 1.40	0.589	7.53 ± 0.66	0.818
	28	3.42 ± 0.01^f^	0.937	3.49 ± 0.16^g^	0.002^d^	7.69 ± 0.98^g^	0.699
***Lpb. plantarum* 5**	0	6.43 ±0.01		0.00 ±0.00		0.00 ±0.00	
	2	4.08 ±0.06^e^	0.002^d^	2.47 ± 0.64^g^	0.002^d^	4.30 ± 0.32^g^	0.002^d^
	14	4.10 ±0.05^f^	0.699	3.95 ± 0.77^g^	0.002^d^	6.20 ± 0.41^g^	0.093
	28	4.20 ±0.05^f^	0.589	4.00 ± 0.59^g^	0.240	5.89 ± 0.18^g^	0.180
***Lpb. plantarum* 9**	0	6.59 ± 0.08		0.00 ± 0.00		0.00 ± 0.00	
	2	3.86 ± 0.07^e^	0.002^d^	3.04 ± 0.89^g^	0.004^d^	6.30 ± 0.32^g^	0.002^d^
	14	3.71 ± 0.17^f^	0.002^d^	3.95 ± 1.24^g^	0.015^d^	6.98 ± 0.76^g^	0.818
	28	3.42 ±0.01^f^	0.240	3.36 ± 0.23^g^	0.004^d^	6.59 ± 0.26^g^	0.937
***Lpb. plantarum* 10**	0	6.36 ± 0.01		0.00 ±0.00		0.00±0.00	
	2	3.94 ± 0.09^e^	0.002^d^	6.80 ± 0.47^g^	0.002^d^	8.14 ± 0.16^g^	0.002^d^
	14	3.90 ± 0.04^**b**^	0.394	7.10 ± 0.78	0.937	7.53 ± 0.66	0.180
	28	3.74 ± 0.10^**b**^	0.009^d^	5.49 ± 0.16^g^	0.132	7.88 ± 0.19^g^	0.065

d Analysis of variance calculated between sampling times for each bacterial strain listed in the same column is considered significant if p < 0.050.

e Analysis of variance calculated at 48 hours between control and per bacterial strain considered significant (p = 0.010).

f Analysis of variance at 14 and 28 days of storage time between control and per bacterial strain (p = 0.010).

g Analysis of variance calculated between lactic acid concentration per strain listed on the same row, presents statistically significance values (p < 0.05).

An increase in the concentration of the lactic acid enantiomers, D-(−) and L-(+)- was detected after 2 days of fermentation (p = 0.002), which was also reflected by the decrease in pH in all the inoculated quinoa-based beverages. Throughout the storage period, the concentrations of D- and L- lactic acid fluctuated. Notably, D-lactic acid concentration primarily decreased, while L-lactic acid concentration increased for some strains by day 28 of storage (Table 2).

The lowest concentration of D-(−)-lactic acid (2.47 ± 0.64 g/L) observed in the beverage inoculated with *Lpb*. *plantarum* 5 after 2 days of fermentation (Table 2) suggests that this strain may have the weakest capacity to inhibit potentially pathogenic bacteria, as viable *Enterobacteriaceae* were also detected. In contrast, the drink inoculated with *Lpb*. *plantarum* 9 also exhibited a lower concentration of D-(−)-lactic acid, but no viable *Enterobacteriaceae* were found (≤ 1). This was not observed in samples inoculated with *Lpb*. *plantarum* 3 and *Lpb*. *plantarum* 10, as both strains demonstrated greater inhibition of *Enterobacteriaceae* and higher concentrations of D-(−)-lactic acid (Table 2). Similar patterns were found for L-(+)-lactic acid. The lowest concentration of L-(+)-lactic acid was found in the samples fermented with *Lpb. plantarum* 5 (4.30 ± 0.32 g/L) (Table 2). The higher concentration of L-(+)-lactic acid compared to D-(−)-lactic acid after 2 days of fermentation suggests the involvement of native microbiota during the initial hours of the process. This is supported by the fact that *Enterococcus* spp. is L-(+)-lactic acid producers, while *Lactiplantibacillus* spp. are known to produce D-(−)-, L-(+)-, or DL-lactic acid (Vitetta, Coulson, Thomsen, Nguyen, & Hall, 2017).

### 3.3 Analysis of secondary metabolites

To assess the functionality of the fermented quinoa-based beverage, our initial approach involved employing HPLC/DAD to quantify the total content of bioactive compounds at different stages of fermentation and storage. In the initial state, the quinoa-based beverage exhibited an average concentration of total phenolics of 120.4 ± 9.3 mg/100 g and total flavonoids of 54.9 ± 1.7 mg/100 g (Table S1). The fermentation processes led to a slight increase in total phenolic metabolites for all strains used. However, these concentrations, expressed as mg/L equivalent of gallic acid, mostly showed less statistically significant difference over time (*p* > 0.05). Only *Lpb. plantarum* 10 presented a statistically significant difference (p > 0.05) (Fig. S1). Furthermore, no statistically significant variations were found in the concentrations of total flavonoids identified at 360 nm using relative quantification with quercetin equivalence (Fig. S1). However, these quantification methods are an estimation of the amounts where similar compounds are assumed to have the same molar absorptivity. Some changes in composition can be missed as the transformation of metabolites can result in products that absorb at the same wavelength. For instance, it was observed that some of the individually identified polyphenols such as p-coumaric acid and rutin varied among some species. However, only a few metabolites (Table S1) could be identified by matching their pure standards with their retention times, while other major peaks could not be identified through simple HPLC/DAD. For the compounds identified with pure standards, their concentrations in the samples were analysed using external calibration curves where the linearity was above 0.99 (range 2 to 200 mg/L) for all compounds (Table S1). The LODs and LOQs ranged between 0.1 and 2.0 (mg/L) and 1 to 5 (mg/L) respectively (Table S1).

**Fig. 2:**
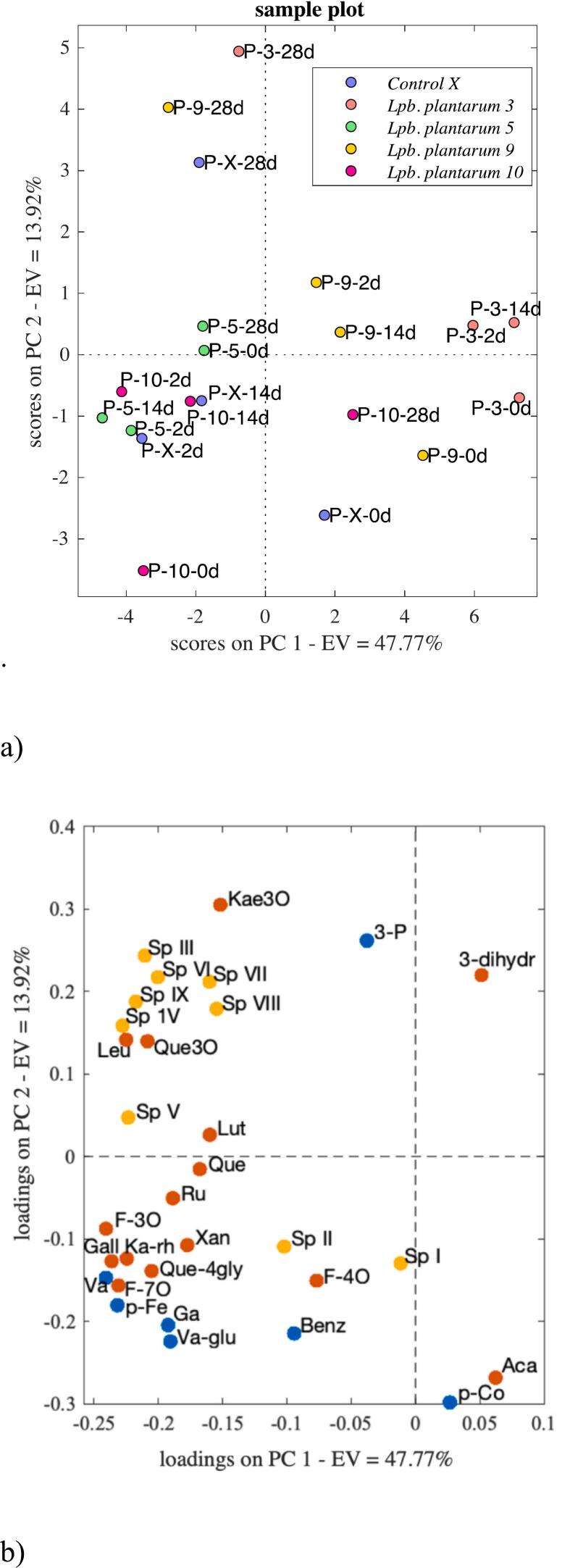
PCA score plot for UHPLC/HRMS data obtained from the quinoa-based beverage fermented with *Lpb. plantarum* 3, 5, 9*;* and 10, and control (X). Score plot of PC1 vs PC2 (a) and loading plot of PC1 vs PC2 (b), classified according to phenolic acids (blue), flavonoids (orange) and saponins (yellow). Compounds full names in Table S2. (For interpretation of the references to colour in this figure legend, the reader is referred to the web version of this article.)

**Fig. 3:**
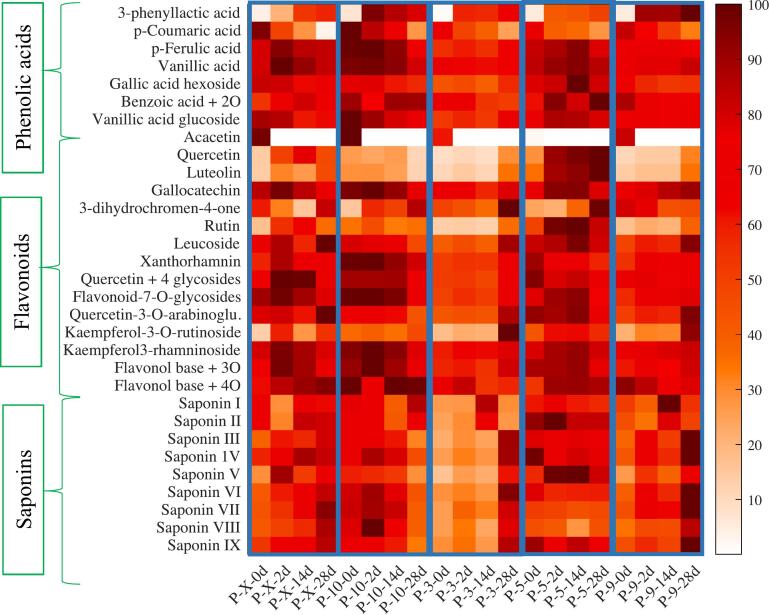
Heatmap plot showing the abundance of each compound after fermentation of the quinoa-based beverage with different *Lpb. plantarum* strains and the control (each separated by the blue lines), as well as changes in compositions according to the fermentation time. The MS intensities in each row were normalized by dividing each with the maximum value obtained and presented as percentage. (For interpretation of the references to colour in this figure legend, the reader is referred to the web version of this article.)

Although ionization inefficiency poses limitations for certain compounds in mass spectrometry, this technique offers notable benefits such as enhanced detection limits, resolution and high confirmation power. Consequently, MS enables the detection of a broader range of compounds compared to the DAD. In this study, further analysis with UHPLC and high-resolution mass spectrometry resulted in annotation of 31 metabolites (Table S2), after deconvolution and library matching in MS-DIAL. Many of the detected and abundant phenolic compounds were flavonoids in their glycosylated forms, a result consistent with outcomes from Gomez-Caravaca et al. (Gómez-Caravaca et al., 2011). Principal component analysis (PCA) revealed separation among *Lpb. plantarum* strains based on the abundancies of the annotated phenolics and saponins (Fig. 2). The PC1 and PC2 accounted for 62 % of the total explained variance. *Lpb. plantarum* 3 and *Lpb. plantarum* 9 were mainly separated from other species along PC1 (48 % variance), mainly due to differences in abundance of compounds. A heatmap illustrated trends in compound abundance (Fig. 3), indicating that the abundance of most compounds increased during fermentation and 14 days of storage, then decreased or remained constant until 28 days. Therefore, samples located on the left side of PC1 correlated to compounds with higher abundance in the loading plots which could illustrate the release of mostly glycosylated flavonoids and phenolic acids during 0–14 days. This trend was observed among the control, *Lpb. plantarum* 5 and *Lpb. plantarum* 10, while the abundance of flavonoids and phenolic acids was relatively low and mostly constant during 0–14 days for *Lpb. plantarum*. 3 and *Lpb. plantarum* 9. Nevertheless, the abundance of many glycosylated flavonoids increased at 28 days of fermentation with *Lpb. plantarum*. 3 and *Lpb. plantarum* 9, contrary to what was observed with other bacterial strains. In general, the changes related to glycosylated flavonoids content with *Lpb. plantarum* 5 and *Lpb. plantarum* 10 showed little difference from the control. Hence this could support the hypothesis of the effect of the native microbiota during fermentation.

Similar phenomena of an increase in flavonoid abundance during the early days of fermentation, followed by a decrease, was also reported by Wang and co-authors (X. Wang et al., 2022). This could be associated with the action of enzymes like oxidoreductases, methyltransferases, glycosyltransferases, etc., which may change phenolic compounds to other forms (Zhao et al., 2019). PC2 exhibited 14 % variation, which mainly separated samples according to fermentation time, where samples after 28 days were separated from 0 to 14 days (Fig. 2 & Fig.S3). Flavonoids clustered in the lower quadrant of PC2, while above it was mainly saponins in the loading plot (Fig. 2). This could suggest that the release of saponins increased mostly with fermentation and storage time, especially among the control, *Lpb. plantarum* 3 and *Lpb. plantarum* 9, although saponins indicated relatively low abundance between 0 and 14 days with *Lpb. plantarum* 3 and *Lpb. plantarum* 9, compared to the control. An increase of saponins during the fermentation of quinoa was also observed by Starzynska-Janiszewska et al. (Starzyńska-Janiszewska et al., 2023), although the authors used fungus for fermentation. Phenolic compounds such as 3-phenyllactic acid, also increased drastically among all the species with fermentation and storage, while some compounds such as p-coumaric and acacetin decreased, which suggests their negative correlation along PC2. 3-Phenyllactic acid is one of the phenolic acids commonly found in fermented foods produced by lactic acid bacteria (Mu, Yu, Zhu, Zhang, & Jiang, 2012). On the contrary, *Lpb. plantarum* 10 showed a decrease in the abundance of several saponins at day 28. The variation along PC3 (12 % variance), with *Lpb*. *plantarum* 10 and *Lpb*. *plantarum* 5 positioned in opposite quadrants (Fig. S4), correlated to the substantial saponin levels observed between 0 and 14 days for *Lpb*. p*lantarum* 10. In contrast, *Lpb*. *plantarum* 5 exhibited a more pronounced increase in aglycone concentrations over 28 days. Saponins are considered anti-nutritional, with high levels (exceeding 860 mg/100 g) potentially causing bitterness (Alrosan et al., 2023). However, certain bacteria are known to facilitate the degradation of saponins.

Previously, it has also been reported that *Lpb. plantarum* spp. favours the formation of aglycone due to promotion of β-glucosidase activities (Landete, Curiel, Rodríguez, de Las Rivas, & Muñoz, 2014). Interestingly, in the present work, not many aglycone were released after the 28 days of storage. However, *Lpb. plantarum* 5 showed a unique decrease in glycosylated flavonoids while aglycones such as luteolin, quercetin, gallocatechin and rutin increased in abundance over time.

As the polyphenol profile resulting from fermentation by lactobacilli was shown to be highly diverse, controlling fermentation time could be essential for promoting the release of desired phenolic compounds. For instance, Li et al. (Li et al., 2020) reported an increase in the content of phenolic acids after 48 h of fermentation of soya, which the authors mainly associated with hydrolysis of polyphenolic compounds into simple phenolics. On the other hand, some phenolic compounds may be degraded by enzymatic actions, especially favoured by increased fermentation time as observed by Alrosan and co-workers (Alrosan et al., 2023). However, it should be noted that the outcomes in phenolic content after fermentation using lactic acid bacteria are complex as they depend on the starter culture, fermentation time as well as the substrate.

While the correlation between phyla and the composition of secondary metabolites was not immediately apparent, it is possible that the observed changes in Proteobacteria and Firmicutes, the dominant phyla, contribute to the distinct concentrations of phenolic compounds. This occurs because the distinct microbial patterns introduced by different starter cultures during quinoa-based beverage fermentation can activate specific metabolic pathways, leading to the release of diverse compounds. Quinoa-based beverages fermented by *Lpb. plantarum* 3, *Lpb. plantarum* 9 and *Lpb. plantarum* 10 as starter cultures were mainly dominated by Firmicutes, in comparison to the control, where Proteobacteria was dominant at the beginning of the fermentation. These starter cultures also commonly show less release of aglycone flavonoids such as quercetin and luteolin. *Lpb. plantarum* 10, showing one of the highest dominances of Firmicutes, could be related to some metabolic activities that contributed to decreased saponins during fermentation. Despite that the use of *Lpb. plantarum* 5 promoted the release of aglycones, the resulting beverage also constituted approximately 40 % of the Proteobacteria phyla (Fig. 1). Proteobacteria are less desirable as they are known to contain potential pathogenic taxa, that also induce different enzymatic activities modifying the biochemical compositions. Polyphenols have additionally been reported to favour the growth of beneficial bacteria (Liu et al., 2020) and counteract fungi (von Staszewski, Pilosof, & Jagus, 2011). Nonetheless, the reduction in pH could also play an effective role in inhibiting pathogenic bacteria, while healthy bacteria like Firmicutes can adapt and grow in this low pH environment. On the other hand, the antimicrobial activity of phenolic acids has been reported in previous studies (Sánchez-Maldonado, Schieber, & Gänzle, 2011).Therefore, the number of live bacterial cells, the observed decrease in pH (≤ 4), and the changes in the composition of secondary metabolites after fermentation, such as polyphenolic compounds, are dependent on the efficiency of the starter culture for the particular substrate. However, other factors involved during fermentation and pretreatment, such as fermentation time, temperature and water content are also essential in the observed chemical and microbial patterns.

## 4 Conclusion

This study investigated the effects of fermentation with different bacterial species on the microbial composition and secondary metabolite profiles of fermented quinoa-based beverages. The study found that the native microbiota significantly influences the chemical composition of the fermented quinoa-based beverage, while starter cultures vary in their contribution to the product's hygiene and safety. A more comprehensive conclusion could be reached by tracking individual metabolite trends rather than relying on total phenolic quantification. For instance, total phenolics using gallic acid equivalence quantification presented insignificant differences between strains, while some profiles of individual compounds were distinct among strains. Some distinctive patterns, dependent on the starter culture, were observed, leading to biochemical changes different from the control. Other species showed an increase in the abundance of phenolic compounds during the first 2 days of fermentation and up to 14 days of storage, followed by a reduction at 28 days. In contrast, *Lpb*. *plantarum* 3 and *Lpb*. *plantarum* 9 exhibited a continued increase in phenolic compound abundance at 28 days. *Lpb. plantarum* 5 increased aglycone abundance, while *Lpb. plantarum* 10 decreased the saponin content compared to the control. Despite variations in secondary metabolites, *Lpb. plantarum* 3, *Lpb. plantarum 9,* and *Lpb. plantarum* 10 showed potential as inhibitors of pathogens. The study acknowledges limitations, including unidentified secondary metabolites and the lack of reference standards. Nonetheless, the findings provide valuable insights for selecting appropriate starter cultures in the production of fermented quinoa-based beverages, thereby influencing consumer safety, functionality and desired metabolite profiles.

### 4.1 Registration of bacterial strains

A stock of the *Lactiplantibacillus plantarum* strains is protected under the Budapest Treaty in the Belgian Coordinate Collections of Microorganisms (BCCM), Laboratorium voor Microbiologie-Bacterienverzameling (LMG), patent collection. The following ascension numbers have been assigned: *Lactiplantibacillus plantarum* LMG P-31891 (3), *Lactiplantibacillus plantarum* LMG P-31894 (5), *Lactiplantibacillus plantarum* LMG P-31893 (9) and *Lactiplantibacillus plantarum* LMG P-31892 (10).

## Funding

This work was financially supported by the Swedish International Development Cooperation Agency (SIDA) [Funding number ID 75000553] as the primary funder, and the Swedish Research Council FORMAS (2018–01863). Lund University funded the APC.

## CRediT authorship contribution statement

**Pamela Canaviri-Paz:** Writing – review & editing, Writing – original draft, Visualization, Validation, Methodology, Investigation, Data curation, Conceptualization. **Thamani Freedom Gondo:** Writing – review & editing, Writing – original draft, Visualization, Validation, Methodology, Investigation, Formal analysis, Data curation. **Anna Kjellström:** Validation, Formal analysis. **Tawanda Mandoga:** Investigation. **Jaison Sithole:** Investigation. **Elin Oscarsson:** Writing – review & editing, Supervision, Methodology, Formal analysis. **Margareta Sandahl:** Writing – review & editing, Visualization, Supervision, Project administration. **Åsa Håkansson:** Writing – review & editing, Visualization, Supervision, Project administration.

## Declaration of competing interest

The authors declare that they have no known competing financial interests or personal relationships that could have appeared to influence the work reported in this paper.

## Data Availability

Data will be made available on request.

## Appendix A Supplementary data

Supplementary material Image 1
