# Supplementary material for: Influence of autochthonous Lactiplantibacillus plantarum strains on microbial safety and bioactive compounds in a fermented quinoa-based beverage as a non-dairy alternative
Source: Food Chem X. 2025 Feb 21;26:102294. doi: 10.1016/j.fochx.2025.102294 (PMC11914179; doi:10.1016/j.fochx.2025.102294)
Supplement: Supplementary file 1 — Supplementary material [file mmc1.docx]

Supporting Information:

**Influence of autochthonous *Lactiplantibacillus* *plantarum* strains on microbial safety and bioactive compounds in a fermented quinoa-based beverage as a non-dairy alternative**

Pamela Canaviri-Paz ^1*^, Thamani Freedom Gondo^2*^, Anna Kjellström^1^, Tawanda Mandoga^1^, Jaison Sithole^1^, Elin Oscarsson^1^, Margareta Sandahl^2^ and Åsa Håkansson^1^.

^1^Department of Process and Life Science Engineering. Faculty of Engineering, LTH, Lund University. Box 124. SE-221 00. Lund, Sweden.

^2^Centre for Analysis and Synthesis. Department of Chemistry. Faculty of science. Lund university. Box 124. 221 00. Lund-Sweden.

^*^Both corresponding authors contributed equally to the experimental part of this paper.

[thamani_freedom.gondo@chem.lu.se](mailto:thamani_freedom.gondo@chem.lu.se)

[pcanaviripaz41@gmail.com](mailto:pcanaviripaz41@gmail.com)

Starter culture reactivation and concentration…………………………………………………2

[Table S1: External standards with the corresponding retention time (Rt), the limit of detection (LOD), limit of quantification (LOQ), coefficient of determination (R^2^) and amount quantified with HPLC/DAD in the fermented quinoa-based beverage used as control (X). 3](#_Toc187603067)

[Table S2:Tentative secondary metabolites in the fermented quinoa-based beverage detected by UHPLC/HRMS and annotated by MS-DIAL. 6](#_Toc187603068)

[Figure S1: Quantification of total phenolics at 280 nm (A) and total flavonoids at 360 nm (B), expressed as mg-equivalent of gallic acid/100g and mg-equivalent of quercetin/100g of fermented quinoa-based beverage (FQB), respectively. 4](#_Toc188539675)

[Figure S2: Chromatogram of quinoa samples after HPLC/DAD analysis, showing phenolic compounds recorded at 280 nm. 5](#_Toc188539676)

[Figure S3: PCA score plot for UHPLC/HRMS data obtained from quinoa-based drink fermented with four *Lactiplantibacillus plantarum* strains and the control (X). 9](#_Toc188539677)

[Figure S4: PCA score plot for UHPLC/HRMS data obtained from a quinoa-based drink fermented with four *Lactiplantibacillus plantarum* species and the control (X). 10](#_Toc188539678)

**Starter culture reactivation and concentration**

Bacterium strains used as starter cultures were isolated from quinoa grains on a previous study at Lund University and stored at -80 °C on freezing media (Canaviri-Paz et al., 2021). 50 µL from the frozen stock were transferred to 5 mL of Man Rogosa and Sharpe broth (MRS broth. Merck. Germany) and incubated at 37 °C overnight (18 h max). The multiplied cells were washed with sterile Milli-Q water (autoclaved at 121 °C. 15 min) and centrifuged at 4266×g for 5 minutes. The cells were reconstituted in 1 mL autoclaved water (121 °C for 15 min) and used as inoculum. Starer culture concentration was measured spectrophotometrically at 610 nm. The starter culture concentration was expressed as Log10 cfu/mL(Nigatu et al., 2000).

Table S1: External standards with the corresponding retention time (Rt), the limit of detection (LOD), limit of quantification (LOQ), coefficient of determination (R^2^) and amount quantified with HPLC/DAD in the fermented quinoa-based beverage used as control (X).

| **Phenolics** | **mg/mL** | | | **Rt** | **Equation of the line** | **R^2^** | **Concentration in Quinoa sample mg/100g**  **(n = 3)** |
| --- | --- | --- | --- | --- | --- | --- | --- |
|  | **LOD** | | **LOQ** |  |  |  |  |
| Gallic acid | 0.82 | 2.49 | | 3.291 | y = 54.604x – 54.464 | 0.998 | ND |
| 3,4-dihydroxybenzoic acid | 0.90 | 2.73 | | 5.674 | y = 31.89x – 55.477 | 0.998 | ND |
| Catechin | 2.07 | 6.26 | | 7.745 | y = 3.4405x – 1.937 | 0.996 | ND |
| 4-hydroxybenzoic acid | 0.32 | 0.98 | | 8.76 | y = 48.934x – 44.914 | 0.999 | ND |
| Vanillic acid | 0.20 | 0.61 | | 10.318 | y = 41.403x – 332.68 | 0.993 | 2.09±0.11 |
| Syringic acid | 0.11 | 0.33 | | 11.218 | y = 64.884x -7.0665 | 0.999 | ND |
| Chlorogenic acid | 0.84 | 2.53 | | 10.317 | y = 1.046x – 1.6406 | 0.998 | ND |
| Caffeic acid | 0.25 | 0.76 | | 10.712 | y = 1.001x + 2.0212 | 1.000 | ND |
| p-Coumaric acid | 0.96 | 2.92 | | 14.846 | y = 105.16x – 206.94 | 0.999 | 1.06±0.06 |
| Ferulic acid | 0.28 | 0.85 | | 16.222 | y = 110.34x – 375.37 | 0.999 | 2.14±0.02 |
| Trans-cinnamic acid | 2.84 | 8.61 | | 24.406 | y = 153.64x – 276.05 | 0.993 | ND |
| Total phenolics (280nm) GAE |  |  | |  |  |  | 120.4±.9.3 |
| **Flavonoids** | | | | | | |  |
| Epicatechin | 5.85 | 17.1 | | 12.336 | y = 1.3989x – 11.243 | 0.992 | ND |
| Rutin | 0.40 | 1.23 | | 17.106 | y = 34.65x – 393.62 | 0.989 | 2.89±0.11 |
| Quercetin 3O-glucoside | 0.13 | 0.39 | | 17.894 | y = 33.947x – 101.22 | 0.997 | ND |
| Mycetrin | 5.90 | 17.8 | | 20.929 | y = 58.343x – 840.3 | 0.995 | ND |
| Quercetin | 0.93 | 2.81 | | 25.248 | y = 69.01x – 460.22 | 0.999 | 1.48±0.01 |
| Kaempferol | 3.35 | 10.1 | | 25.761 | y = 52.105x – 266.33 | 0.987 | ND |
| Total flavonoids (360nm) QAE |  |  | |  |  |  | 54.9±1.7 |

GAE: Gallic acid equivalence.

QAE: Quercetin acid equivalence.

ND: Not detected/below LOQ

Figure S1: Quantification of total phenolics at 280 nm (A) and total flavonoids at 360 nm (B), expressed as mg-equivalent of gallic acid/100g and mg-equivalent of quercetin/100g of fermented quinoa-based beverage (FQB), respectively.


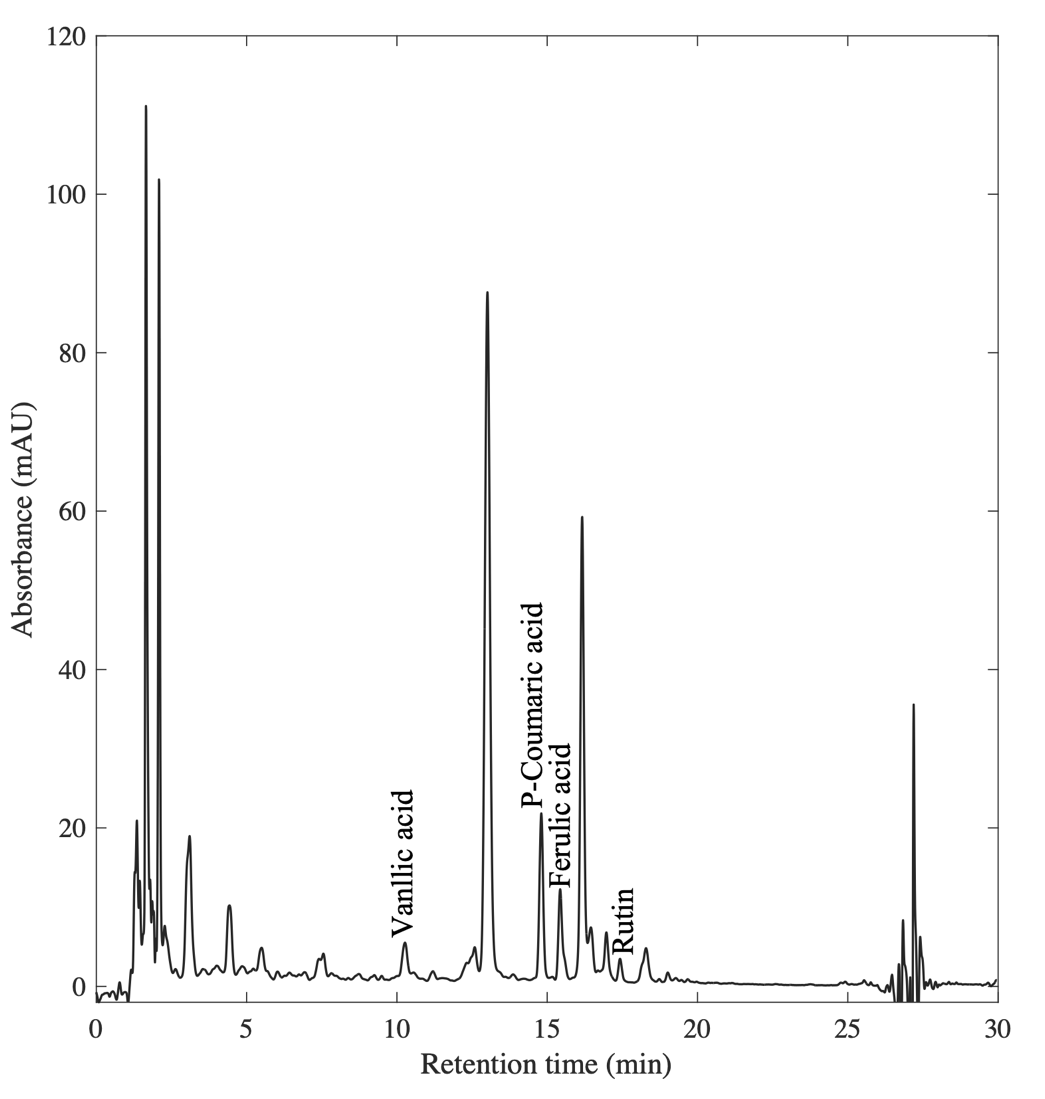


Figure S2: Chromatogram of quinoa samples after HPLC/DAD analysis, showing phenolic compounds recorded at 280 nm.

The peaks which are not annotated were not identified with available reference standards.

Table S2:Tentative secondary metabolites in the fermented quinoa-based beverage detected by UHPLC/HRMS and annotated by MS-DIAL.

Classes of compounds are noted as ph, fv and sp for phenolic acids, flavonoids and saponins respectively.

| **No.** | **RT**  **UHPLC-MS** | **Compound name** | **Abbr.** | **Class** | **Exact mass (M-H)** | **Observed mass**  **(M-H)** | **Mass difference**  **(mDa)** |
| --- | --- | --- | --- | --- | --- | --- | --- |
|  | 1.05 | Acacetin | Aca | Fv | 283.06847 | 283.06421 | 4.26 |
|  | 3.78 | Benzoic acid + 2O (beta-D-Xylopyranose, 1-(3,4-dihydroxybenzoate) | Benz | Ph | 285.06887 | 285.06088 | 7.99 |
|  | 3.99 | Gallic acid hexoside | Ga | Ph | 331.07435 | 331.0704 | 3.95 |
|  | 4.63 | Quercetin + 4 glycosides (3',4',5,7-Tetrahydroxy-3-[[2-O-(3-O-beta-D-glucopyranosyl-alpha-L-rhamnopyranosyl)-6-O-alpha-L-rhamnopyranosyl-beta-D-galactopyranosyl]oxy]flavone) | Que-4gly | Fv | 917.26412 | 917.25818 | 5.94 |
|  | 4.75 | Vanillic acid | Va | Ph | 167.04226 | 167.03624 | 6.02 |
|  | 4.91 | 3-Phenyl lactic acid | 3-P | Ph | 165.06299 | 165.05388 | 9.12 |
|  | 4.93 | Flavonol base + 3O glycoside (Kaempferol-3-O-glucosyl-rhamnosyl-glucoside) | F-3O | Fv | 755.21129 | 755.20142 | 9.88 |
|  | 5.04 | Flavonol base + 4O glycoside (kaempferol 3-O-β-D-apiofuranosyl(1'''-> 2''- O-[α-L-rhamnopyranosyl (1'''-> 6'' ]-β-D-galactopyranoside) | F-4O | Fv | 741.19565 | 741.18457 | 11.08 |
|  | 5.33 | Quercetin-3-O-arabinoglucoside | Que3O | Fv | 595.13774 | 595.12799 | 9.75 |
|  | 5.41 | Kaempferol-3-rhamninoside | Ka-rh | Fv | 739.21638 | 739.20734 | 9.04 |
|  | 5.57 | Flavonoid-7-O-glycosides (1,2 -dihydroxyreruloyl-gentiobiose) | F-7O | Fv | 725.20073 | 725.19482 | 5.91 |
|  | 5.66 | Xanthorhamnin | Xan | Fv | 769.22695 | 769.22534 | 1.61 |
|  | 5.73 | p-Coumaric acid | p-Co | Ph | 163.04734 | 163.03828 | 9.07 |
|  | 5.78 | Flavonol base + 3O ( Chrysoeriol-7-O-apiosyl-glucoside) | F-3O | Fv | 593.15847 | 593.14899 | 9.48 |
|  | 5.86 | p-Ferulic acid | P-Fe | Ph | 193.05791 | 193.05093 | 6.98 |
|  | 5.95 | Leucoside | Leu | Fv | 579.14282 | 579.13739 | 5.44 |
|  | 5.99 | Rutin | Ru | Fv | 609.15339 | 609.14697 | 6.42 |
|  | 6.84 | Gallocatechin | Gall | Fv | 305.07395 | 305.07193 | 2.03 |
|  | 7.73 | Quercetin | Que | Fv | 301.04265 | 301.03345 | 9.20 |
|  | 7.85 | Vanillic glucoside | Va-glu | Fv | 329.09508 | 329.0889 | 6.18 |
|  | 7.94 | Saponin I (Azukisaponin VI) | Sp I | Sp | 1133.54582 | 1133.54346 | 2.36 |
|  | 8.08 | Saponin II (3-O-β-D-glucopyranosyl-(1->3)-β-D-galactopyranosyl phytolaccagenic acid 28-O-β-D-glucopyranosyl ester) | Sp II | Sp | 1001.50356 | 1001.50684 | 3.28 |
|  | 8.10 | Saponin III (3-O-α-L-arabinopyranosyl-(1 -> 3)-β-D-glucuronopyranosyl serjanic acid 28-O-β-D-glucopyranosyl ester) | Sp III | Sp | 969.47735 | 969.47467 | 2.68 |
|  | 8.13 | Luteolin | Lut | Fv | 285.04774 | 285.04083 | 6.91 |
|  | 8.15 | Saponin 1V (3-O-β-D-xylopyranosyl-(1->3)-β-D-glucuronopyranosyl 24.7 hederagenin 28-O-β-D-glucopyranosyl ester ) | Sp IV | Sp | 941.48243 | 941.48798 | 5.54 |
|  | 8.47 | Saponin V (3-O-a-L-arabinopyranosyl serjanic acid 28-O-b-D-glucopyranosyl ester) | Sp V | Sp | 793.44526 | 793.43768 | 7.58 |
|  | 8.57 | Saponin VI (3-O-β-D-glucopyranosyl-(1->3)-α-larabinopyranosyl 23.6 phytolaccagenic acid 28-O-β-D-glucopyranosyl ester ) | Sp VI | Sp | 971.49299 | 971.48859 | 4.41 |
|  | 8.58 | Saponin IX ((2S,3S,4S,5R,6R)-6-[[(3S,6aR,6bS,8aS,14bR)-8a-carboxy-4- (hydroxymethyl)-4,6a,6b,11,11,14b-hexamethyl- 1,2,3,4a,5,6,7,8,9,10,12,12a,14,14a-Tetradecahydropicen-3-yl]oxy]-3,4,5- trihydroxyoxane-2-carboxylic acid) | Sp IX | Sp | 647.38735 | 647.38617 | 1.18 |
|  | 8.79 | Saponin VII (2-O-Acetyl-21-O-(2-methylpropanoyl) barrigenol R1 (1-> 4 (3b,15a,16a,21b,22a)-22-(Acetyl- oxy)-3,15,16,28-tetrahydroxyolean-12-en-21-yl 2-methylpropanoate) | Sp VII | Sp | 955.49808 | 955.49835 | 0.27 |
|  | 8.99 | Saponin VIII (3b-[(O-b-D-glucopyranosyl-(1->3)-a-L-arabinopyranosyl)oxy]-27- oxo-olean-12-en-28-oic acid b-D-glucopyranoside) | Sp VIII | Sp | 925.48752 | 925.48584 | 1.68 |
|  | 10.7 | (2R)-7-hydroxy-8-(2-hydroxyethyl)-5-methoxy-2-methyl-2.3-dihydrochromen-4-one | 3-dihyr | Fv | 251.09977 | 251.09586 | 3.92 |

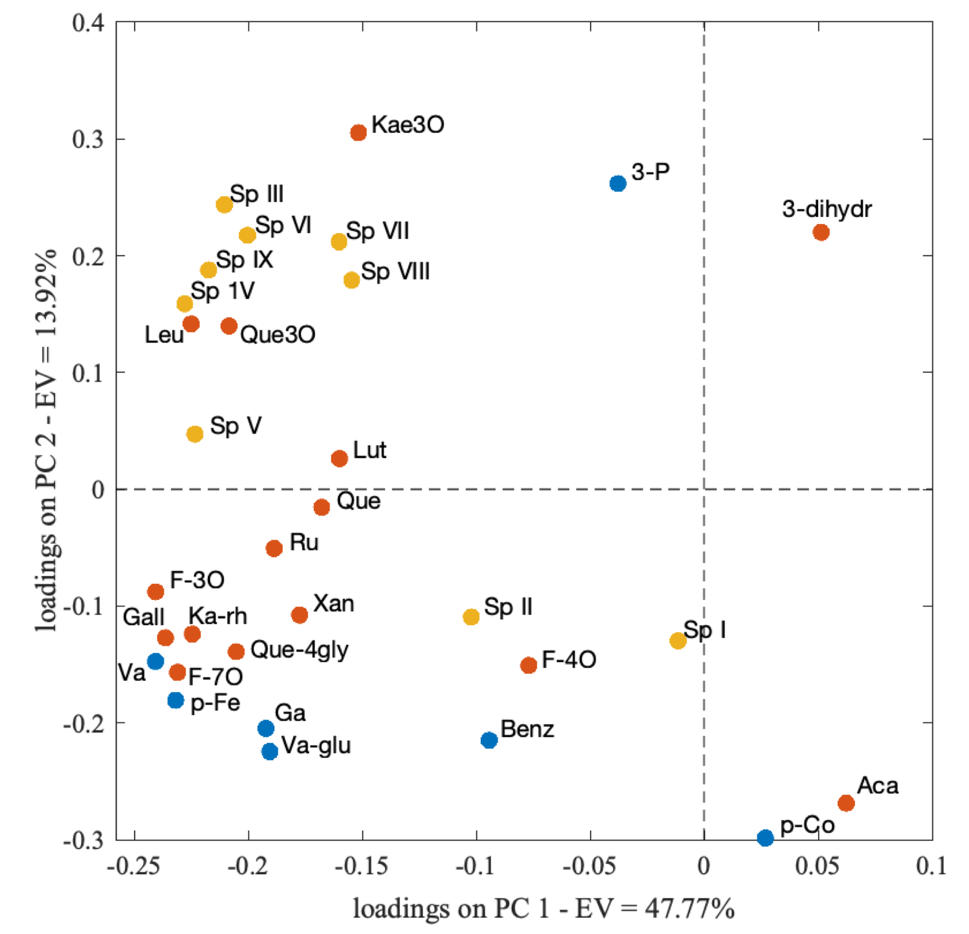


Figure S3: PCA score plot for UHPLC/HRMS data obtained from quinoa-based beverage fermented with four *Lactiplantibacillus plantarum* strains and the control (X).

Score plot of PC1 vs PC2 with samples coloured according to time (top) and loading plot of PC1 vs PC2 (bottom)*,* classified according to phenolic acids (blue), flavonoids (orange) and saponins (yellow). Labels on the score plot indicate samples obtained from different species, P-3, P-5, P-9 and P-10 represent *Lpb. plantarum* 3,5 9 and 10 respectively.

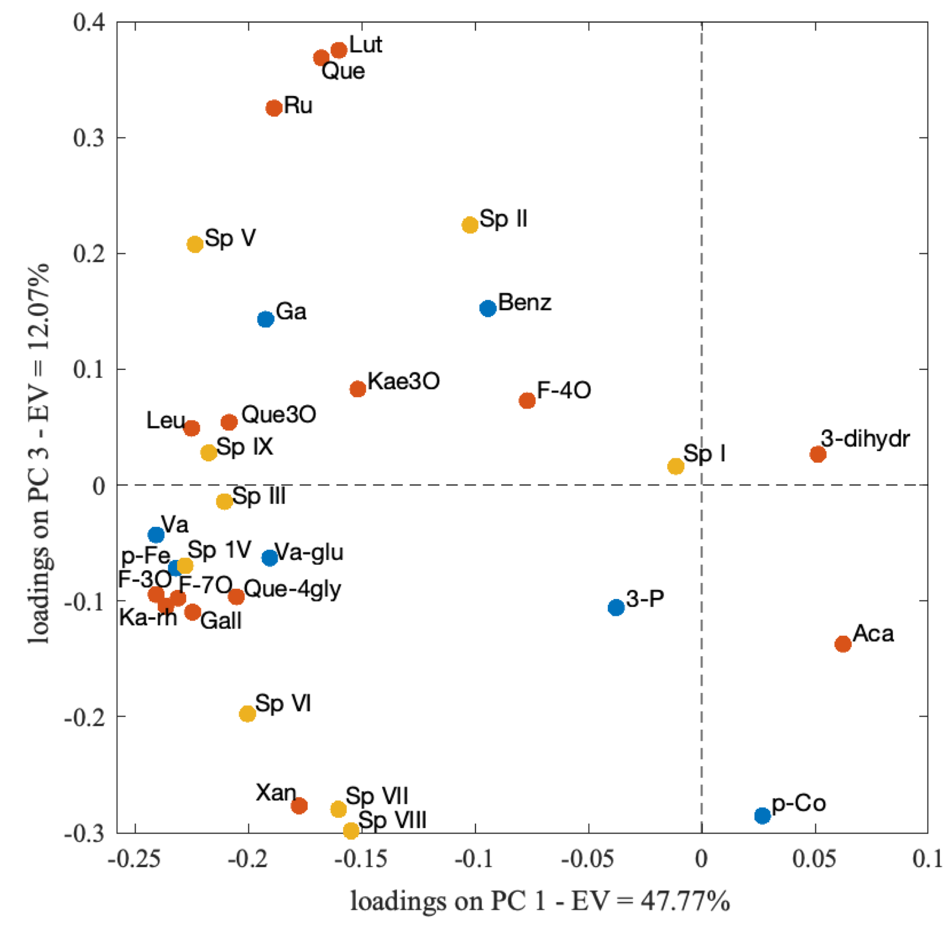


Figure S4: PCA score plot for UHPLC/HRMS data obtained from a quinoa-based beverage fermented with four *Lactiplantibacillus plantarum* species and the control (X).

Score plot of PC1 vs PC3 (top) and loading plot of PC1 vs PC3 (bottom)*,* classified according to phenolic acids (blue), flavonoids (orange) and saponins (yellow).

# **References**

Canaviri-Paz, P., Oscarsson, E., & Håkansson, Å. (2021). Autochthonous microorganisms of white quinoa grains with special attention to novel functional properties of lactobacilli strains. *Journal of Functional Foods*, *84*(March), 104586. https://doi.org/10.1016/j.jff.2021.104586

Nigatu, A., Ahrné, S., & Molin, G. (2000). Temperature-dependent variation in API 50 CH fermentation profiles of Lactobacillus species. *Current Microbiology*, *41*(1), 21–26. https://doi.org/10.1007/s002840010085
